# Usefulness of routine preoperative testing in a developing country: a prospective study

**DOI:** 10.11604/pamj.2015.21.94.5860

**Published:** 2015-06-05

**Authors:** Julien Bordes, Pierre-Julien Cungi, Pierre-Henry Savoie, Stéphane Bonnet, Eric Kaiser

**Affiliations:** 1 Département d'Anesthésie-Réanimation, HIA Sainte Anne, Toulon, France; 2 Service d'Urologie, HIA Sainte Anne, Toulon, France; 3 Service de Chirurgie Viscérale, HIA Percy, Paris, France

**Keywords:** Diagnostic tests, HIV Infections, preoperative period, surgical Procedures

## Abstract

**Introduction:**

The assessment of anesthetic risks is an essential component of preoperative evaluation. In developing world, preanesthesia evaluation may be challenging because patient's medical history and records are scare, and language barrier limits physical examination. Our objective was to evaluate the impact of routine preoperative testing in a low-resources setting.

**Methods:**

Prospective observational study performed in a French forward surgical unit in Abidjan, Ivory Coast. 201 patients who were scheduled for non urgent surgery were screened with routine laboratory exams during preoperative evaluation. Changes in surgery were assessed (delayed or scheduled).

**Results:**

Abnormal hemoglobin findings were reported in 35% of patients, abnormal WBC count in 11,1% of patients, abnormal platelets in 15,3% of patients. Positive HIV results were found in 8,3% of cases. Routine tests represented 43,6% of changes causes.

**Conclusion:**

Our study showed that in a developing country, routine preoperative tests showed abnormal results up to 35% of cases, and represented 43,5% of delayed surgery causes. The rate of tests leading to management changes varied widely, from 0% to 8,3%. These results suggested that selected tests would be useful to diagnose diseases that required treatment before non urgent surgery. However, larger studies are needeed to evaluate the cost/benefit ratio and the clinical impact of such a strategy.

## Introduction

The assessment of anesthetic risks is an essential component of preoperative evaluation. Preanesthesia evaluation aims to identify potential problems that can affect perioperative management or outcome. This evaluation is based on multiple sources, as patient's medical records, interview, physical examination, and laboratory testing. However, studies have shown that systematic preoperative tests were unuseful and should not be ordered routinely [1, 2]. Practice advisory for preanesthesia evaluation are based on studies performed in developed countries [2]. In developing world, preanesthesia evaluation may be challenging because patient's medical history and records are scare, and language barrier limits patient interview. Moreover, diseases which impact postoperative outcome are endemic in some countries, as human immunodeficiency virus (HIV) infection. In Ivory Coast, the prevalence of HIV infection has been reported to be of 15, 83% in rural area [3]. The major risk is to schedule for a non urgent surgery a patient having an undiagnosed disease which may worsen perioperative outcome, or medical problems which require a treatment before surgery. Unfortunately, there are few data on the practice of preanesthesia evaluation and the usefulness of routine preoperative tests in low-ressource settings. That's why we performed a prospective study in Abidjan, Ivory Coast, to evaluate the usefulness of routine preoperative tests.

## Methods

### Location

This study was a prospective study performed in a french military forward surgical unit (FSU) in Abidjan, Ivory Coast, during a 6 months period (from january 2012 to july 2012). The FSU is a light mobile structure permitting anesthesia and surgery closer to combat fields, and working in tents or shelters [4]. It is manned by one general surgeon, one orthopedic surgeon, and one anesthesiologist. In peacetime, the surgical team is allowed to propose a free medical aid to local population during study per.

### Study protocol

Patients from local population were included after oral consent was obtained. Patients who were scheduled for a non urgent surgery were evaluated by an anaesthesiologist after surgical consultation. Preanesthesia evaluation was based on: patient's medical records if available; physical examination; routine laboratory tests. A routine test was defined by the ASA Task Force on Preanesthesia Evaluation as a test ordered in the absence of a specific clinical indication or purpose [2]. Routine tests performed in this study were: hemogram with hemoglobin measurement, white blood cells count, platelets count; human immunodeficiency virus screening; hepatitis C virus (HCV) screening. After preoperative evaluation, the anaesthesiologist decided to confirm or to delay the surgery. The causes of delayed surgery were medical problems that should be treated before the surgery, or diseases that went beyond the FSU medical capacity, and were defined as follow: severe anemia less than 8g/dl; thrombocytopenia less than < 50000 /mm3; neutropenia < 2000/mm3; previously undiagnosed HIV infection with unknown immunological status and previously undiagnosed HCV; non controlled arterial hypertension or coronaropathy; criteria for difficult intubation Patient was addressed to a medical referee is it was judged necessary by the anaesthesiologist. In case of unknown HIV seropositivity, patient was referred to a HIV unit in Abidjan. This was possible because the Ministry of Health/UNAIDS Drug Access Initiative in Ivory Coast was announced in November 1997 and effectively started in August 1998 and facilitated access to HIV specialized care units and treatment [5].

### Data recorded

Patients’ characteristics (age, gender), types of scheduled surgeries, routine tests values, and preoperative decision (confirmed or delayed surgery) were prospectively recorded. The rate of abnormal tests was calculated by: (number of abnormal tests/number of performed tests) x 100. The rate of tests leading to management changes was calculated by: (number of tests leading to changes/number of tests performed) x 100.

### Statistical analyses

Statistical analyses were performed using XLSTATS software (Addinsoft, Paris, France). Descriptive measures were used to present patients’ characteristics and routine tests data: number, percentage, mean (+/- standard deviation, SD). Data between HIV positive and HIV negative patients were compared using a Mann and Whitney test. No a priori power analysis was conducted because we did not know the exact effect size that we would see. A p value of < 0.05 was required to reject the null hypothesis.

## Results

**Figure 1:**
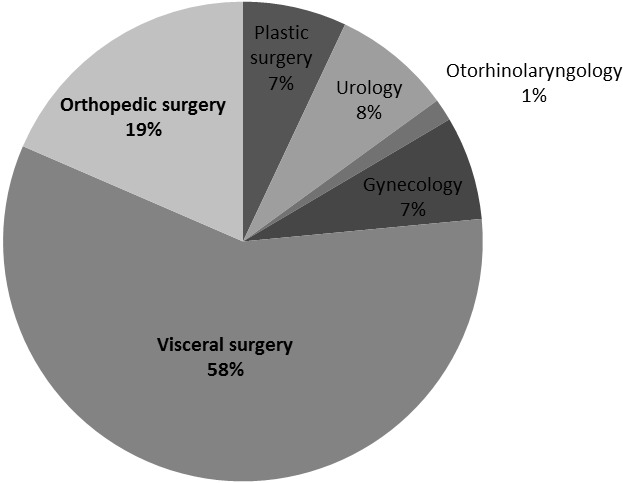
Type of scheduled surgeries

**patients’ characteristics:** two hundred and one patients were included. There were 56 female and 145 male. 58% of patients were scheduled for visceral surgery, 18,5% for orthopedic surgery, 8% for urologic surgery, 7% for gynecologic surgery (Figure 1). The mean age was 39 years old (+/- 17).

**Physical exam's findings:** physical exams showed active infection in one patient, severe poliomyelitis sequelae in one other, difficult airway management criteria in 4 patients, uncontrolled coronary disease in 4 patients, and severe arterial hypertension in 2 patients.

**Hemoglobin measurement:** the mean hemoglobin rate was 12,7 g/dL (+/- 2,3). 29% of patients presented with anaemia (Table 1).

**Table 1:** Preoperative routine tests results

Preoperative tests	Number of performed tests	Normal values	Rate of abnormal tests	Results, mean +/- SD
Hemoglobin, g/dL	117	12-16	35%	12.7 +/- 2.3
WBC count, total, 10^3^/mm^3^	117	4-11	11.1%	5.6 +/- 1.5
-neutrophils,				3.2 +/- 2.8
-lymphocytes,				2.08 +/- 0.7
-monocytes,				0.45 +/- 0.22
-basophils,				0
-eosinophils				0.003 +/- 0.032
Platelets count, 10^3^/mm^3^	117	150-400	15.3%	254 +/- 110
HIV screening	168		8,3%	
HCV screening	57		0%	

**White blood cells count measurement:** the mean white blood cell count was 5.6. 10^3^/mm^3^ (+/- 1.5). Abnormal WBC count was reported in 11,1% of patients (Table 1)

**Platelets count measurement:** the mean platelets count was 254.10^3^/mm^3^. Abnormal platelets count was observed in 15,3% of patients (Table 1).

**Preoperative blood virus screening:** HIV testing was positive in 8.3% of patients. No patient presented with a positive HCV test. Hemoglobin value was significantly lower in patients with a positive HIV test (hb = 10.7g/dL versus 13g/dL, p = 0.0006). However, we did not observe any statistically significant differences between HIV positive and negative patients in term of WBC count, lymphocytes counts and platelets count.

**Preoperative management changes:** preanesthesia evaluation led to management changes in 39 patients (19,4%). Routine tests led to delayed surgery in 17 patients, that represented 43,6% of changes. In other patients, delayed surgeries were due to uncontrolled arterial hypertension (n = 2), uncontrolled coronaropathy (n = 6), and other reasons detailed in Table 2). (n = 14). Hemogram test led to management changes in 1,7% of patients, platelets count in 0,8%, and VIH screening in 8,3% (Table 3).

**Table 2:** Causes of delayed surgery

Delayed patients, n	39
Physical exam, n (%)	18 (46.2%)
Active infection	1 (2.5%)
Uncontrolled coronary disease	6 (15.4%)
Severe arterial hypertension	2 (5.1%)
Difficult airway management criteria	4 (10.2%)
Lack of surgical materials	2 (5.1%)
Hemoglobin value	2 (5.1%)
Platelets count	1 (2.5%)
HIV screening	14 (35.9%)
No detailed causes	7 (17.9%)

**Table 3:** Preoperative routine tests leading to delayed surgery

Tests	n	Rate of tests leading to changes
Hemoglobin value	2	1,7%
Platelets count	1	0,8%
HIV screening	14	8,3%
HCV screening	0	0%

## Discussion

Our study showed that in a developing country, routine preoperative tests showed abnormal results up to 35% of cases, and represented 43,5% of delayed surgery causes. The rate of tests leading to management changes varied widely, from 0% to 8,3%. Preanesthesia evaluation aims to identify potential problems that can affect perioperative management or outcome. Anaesthetic-related mortality has been reported to be two to three times higher in developing countries than in developed countries in a meta-analysis [6]. Other studies suggest that the situation is much worse. The incidence of 24-h perioperative deaths per 100 anaesthetics was 2.57 in a study performed in Togo [7]. In Malawi, it has been estimated that the avoidable mortality rate was 6 to 100 times higher than in developed countries [8]. Moreover, it has been reported that 80% of perioperative caesarean section deaths occur on general wards in the postoperative period [9]. To date, it is not evaluated if preanesthesia evaluation is an avoidable factor of mortality. Practice advisory for preanesthesia evaluation recommend not to order routinely preoperative tests [2]. No data support the pertinence of these recommendations in developing countries. Our results showed hemoglobin was abnormal in 35% of cases. These results were concordant with previoulsy published data in developed countries, showing that in asymptomatic or nonselected patients, abnormal hemoglobin findings were reported in 0.5% to 65.4% of patients [2]. However, the hemoglobin test led to changes in 1.7% of cases, rate which is concordant with other studies [2]. White blood cell count did not led to surgical changes, and platelets count for only one patient. These results support the following points. Routine hemogram before surgery do not make an important contribution to preoperative decision. Hemoglobin is the most pertinent parameter to measure among hemogram parameters. The question of preoperative HIV testing of patients remains controversial. In one hand, a routine testing may be opposed because of the implications of a positive HIV test results and the fear that HIV-positive patients would receive non optimal treatment. In the other hand, HIV screening may be beneficial for the patient, allowing diagnosis of the disease in a early stage, and prompt anti-retroviral therapy. This beneficial effect is increased in countries where the health system allows population to access to HIV treatment, as in Ivory Coast. Indeed, the Ministry of Health/UNAIDS Drug Access Initiative in Ivory Coast was announced in November 1997 and effectively started in August 1998 and facilitated access to HIV specialized care units and treatment [5]. Some data have been published demonstrating that HIV-positive patients had more postoperative complications than HIV-negative patients. In the study of Gruber et al performed in Germany, the overall rate of complications following surgery was substantially increased for HIV-infected patients, compared with patients in the control group (18.7% versus 6.8%) [10]. Again, the « major complications », as infection during the postoperative clinical period and the need to perform additional surgical procedures, occurred more frequently in HIV-infected patients. In this study, multivariate analysis was performed to assess the effect of factors that could have influenced patient outcome. CD4 cell counts

From a public health point of view, the presurgical screen for HIV may be beneficial in endemic country, by participating to fight against the disease. In Ivory coast, the prevalence is estimated from 2292 to 7734 cases per 100000 [11]. The mean prevalence of HIV infection in a study was 5.30% performed in six rural areas, with a peak at 15,833% [3]. Our study showed that in an endemic area, HIV screening may be useful, with a rate of 8,3% of positive tests. Guidelines from the UK′s National Institute for Health and Clinical Excellence suggested screening of all new patients in general practice from populations in which HIV prevalence exceeds two in 1000 [12]. It would also be argued that HIV screening benefits members of the surgical and anesthesia team. Indeed, awareness of HIV status would facilitate application of suitable precautions and strating of postexposure prophylaxis immediately after exposure to a patient′s blood [13]. Our study had limitations. The first limitation is the limited preoperative tests we could perform. This limitation was explained by the limited technical support we had during the study period. Only 58% of enrolled patients had hemogram tests. This point emphasizes the logistical difficulties of a preoperative evaluation based on routine tests in low-ressources setting. Screening test consume considerable patient and physician time, and financial ressources, and may represent the most serious limit. And evaluation of cost-effectiveness of such a strategy would have been interesting. The second limitation is the choice of preoperative tests. In particular, we did not screen glycemia. In a study performed at a tertiary-care referral center in South India, only screening for diabetes seemed to have usefulness as a routine test in the studied population [14]. The third limitation is due to the absence of postoperative follow-up.

## Conclusion

Published data on the perioperative mortality and morbidity in developing countries emphasize the urgent need to find ways to improve outcome. Preoperative evaluation could be one of the components of this improvement. Our study showed that selected routine tests would be useful to diagnose diseases that require treatment before non urgent surgery. However, larger studies are needed to evaluate the cost/benefit ratio and the clinical impact of such a strategy.
